# 3-Dimensional Model to Study Apoptosis Induction of Activated Natural Killer Cells Conditioned Medium Using Patient-Derived Colorectal Cancer Organoids

**DOI:** 10.3389/fcell.2022.895284

**Published:** 2022-05-26

**Authors:** Benyamin Parseh, Ayyoob Khosravi, Abdolreza Fazel, Jafar Ai, Somayeh Ebrahimi-Barough, Javad Verdi, Majid Shahbazi

**Affiliations:** ^1^ Department of Applied Cell Sciences, School of Advanced Technologies in Medicine, Tehran University of Medical Sciences, Tehran, Iran; ^2^ Faculty of Advanced Medical Technologies, Golestan University of Medical Sciences, Gorgan, Iran; ^3^ Stem Cell Research Center, Golestan University of Medical Sciences, Gorgan, Iran; ^4^ Department of Molecular Medicine, Faculty of Advanced Medical Technologies, Golestan University of Medical Sciences, Gorgan, Iran; ^5^ Cancer Research Center, Golestan University of Medical Sciences, Gorgan, Iran; ^6^ Department of Tissue Engineering, School of Advanced Technologies in Medicine, Tehran University of Medical Sciences, Tehran, Iran; ^7^ Medical Cellular and Molecular Research Center, Golestan University of Medical Sciences, Gorgan, Iran

**Keywords:** tumor organoid, colon cancer, apoptosis, NK cells, conditioned medium

## Abstract

Natural killer (NK) cells are innate lymphocytes that can kill tumor cells via different pathways, including the secretion of cytotoxic granules in immunological synapses and the binding of apoptosis-inducing ligands with cognate death receptors on tumor cells. These ligands are also soluble in NK cells conditioned medium (NK-CM). However, novel preclinical *in vitro* models are required for solid tumors such as colorectal cancer (CRC) to investigate apoptosis induction of activated NK-CM in a tissue-like structure. In the present study, we established a patient-derived CRC organoid culture system as a new tool for CRC research in the last decade**.** Tumor organoids were stained with hematoxylin and eosin (H&E) and compared with the original tumor taken from the patient. Goblet cell differentiation and mucus secretion were evaluated using periodic acid–Schiff and alcian blue histochemical staining. Moreover, tumor organoids were stained for CDX2 and Ki67 markers with immunohistochemistry (IHC) to investigate gastrointestinal origin and proliferation. Histopathological evaluations indicated tumor organoids represent patient tumor characteristics. Primary NK cells were isolated and characterized using CD56 marker expression and the lack of the CD3 marker. Flow cytometry results showed the purity of isolated CD3^−^and CD56 ^+^ NK cells about 93%. After further *ex vivo* expansion, IL-2-activated NK-CM was collected. Secretions of IFN-γ and TNF-α were measured to characterize activated NK-CM. Cytokines levels were significantly elevated in comparison to the control group. Soluble forms of apoptosis-inducing ligands, including TNF-related apoptosis-inducing ligand (TRAIL) and FasL, were detected by western blot assay. Colon cancer organoids were treated by IL-2-activated NK-CM. Apoptosis was assessed by Annexin V-FITC/PI staining and quantified by flow cytometry. In conclusion, despite the activated NK-CM containing apoptosis-inducing ligands, these ligands’ soluble forms failed to induce apoptosis in patient-derived colon cancer organoids. Nevertheless, we report a reliable *in vitro* assessment platform in a personalized setting.

## Introduction

Natural killer (NK) cells are effector lymphocytes of the innate immune system against infected or transformed cells (Abdolahi et al., 2021). NK cells express lytic machinery that can kill target cells independently from the previous activation (Zamai et al., 2007). Recruitment of NK cells against various solid tumors is a promising cancer treatment strategy (Gras Navarro et al., 2015). Understanding the fundamental processes driving NK cell detection, stimulation, and suppression allows immunotherapeutic approaches to induce NK cell responses against tumors (Wu et al., 2020). Various studies have shown that using NK cells for solid tumors has several drawbacks. However, NK cell-based immunotherapies have been more efficacious in hematologic cancers (Nayyar et al., 2019; Shin et al., 2020). NK cells exhibit profound degranulation defects in cancer patients. The low infiltration of NK cells in the tumor site is the main challenge against solid tumors. Also, NK cells are vulnerable to various immunosuppressive mechanisms found in the tumor microenvironment (Schnalzger et al., 2019).

NK cells are known for two types of cells killing. The granule exocytosis pathway releases cytotoxic granule components between NK and target cells (granzymes, granulysin, and perforin) into the immunological synapse. Granzymes had essential functions in triggering target cells’ death *via* caspases-independent or dependent pathways (Smyth et al., 2005). Activated NK cells express different types of apoptosis-inducing ligands, such as tumor necrosis factor-α (TNF-α), Fas ligand (FasL), and TNF-related apoptosis-inducing ligand (TRAIL) (Smyth et al., 2002; Aggarwal, 2003). These death ligands are transmembrane proteins that are also proteolytically cleaved and released as a soluble form in NK cells conditioned medium (NK-CM) (Chávez-Galán et al., 2009; Sheard et al., 2013). In another pathway, the binding of FasL, TRAIL, or TNF-α induces cell death *via* death receptors (DRs) on the surface of cancer cells (Diaz Arguello and Haisma, 2021). DRs are members of the TNF receptor superfamily linked to apoptosis signaling independent of the p53 tumor suppressor gene. TNF-R1 and TNF-R2 for TNF-α, Fas for FasL, and DR4/TRAILR1, and DR5/TRAILR2 for TRAIL are the most studied DRs that possess death domain that can induce apoptosis (Diaz Arguello and Haisma, 2021). Activated NK cells are also known to release IFN-γ in the tumor microenvironment, which is necessary for NK cell activity against various cancers (Bald et al., 2020). Also, IFN-γ improves FasL and TRAIL function by sensitizing DRs and increasing TRAIL expression (Wajant et al., 2005). Conversely, cancer cells respond quickly to IFN-γ released by activated NK cells. The signaling of IFN-γ in cancer cells promotes PD-L1 expression, which inhibits NK cytolytic function and subsequent IFN-γ production. This negative regulatory feedback mechanism inhibits the activation of NK cells and leads to tumor survival (Bellucci et al., 2015).

**FIGURE 1:**
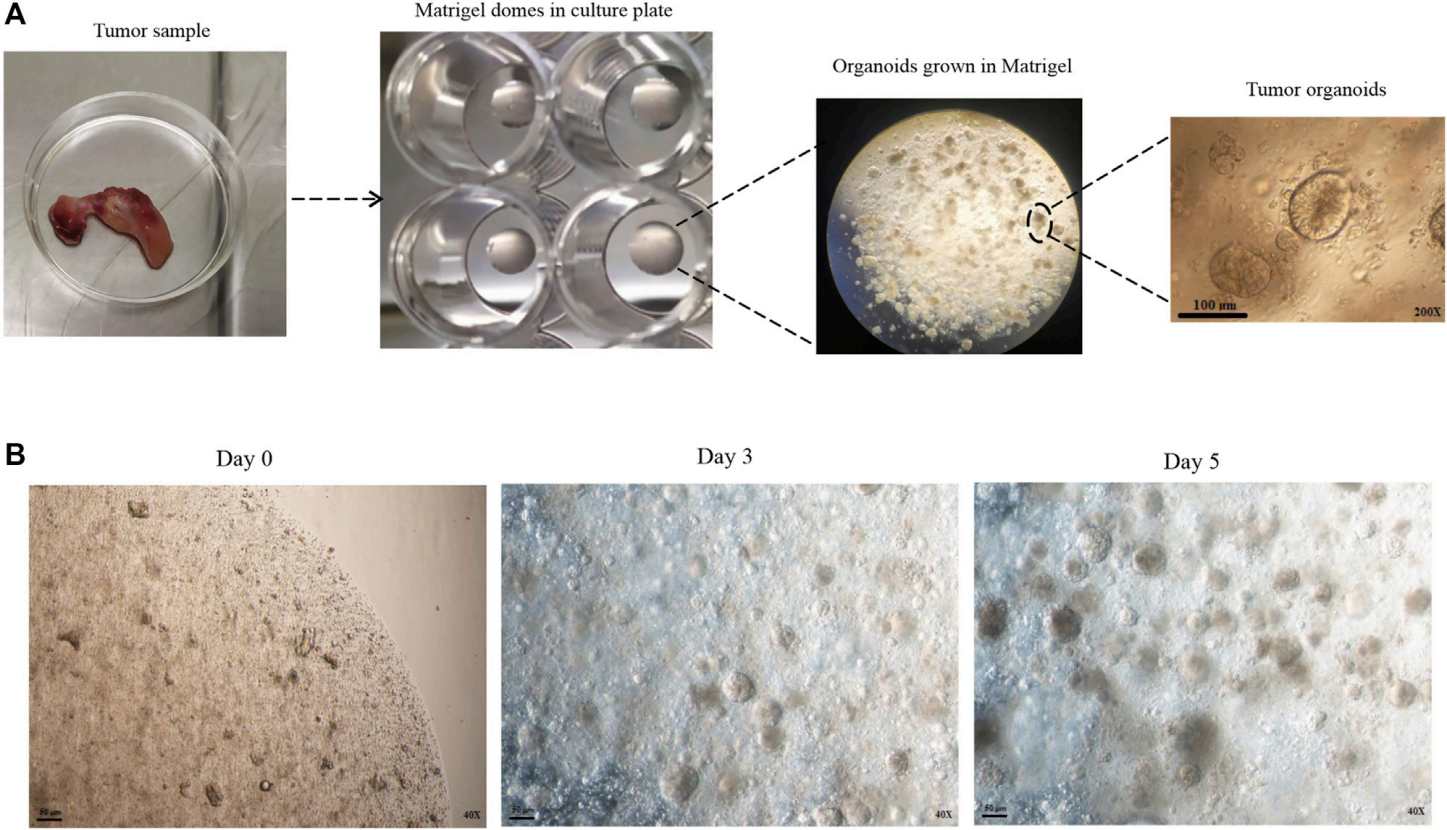
Development of patient-derived colon cancer organoid. **(A)** Tumor samples were taken from the patient. After tissue digestion, the cells and crypts were seeded with Matrigel in a 48 well plate. After adding the organoid culture medium to the Matrigel dome, the three-dimensional structure of the organoids is formed. **(B)** Different time intervals of organoids in the culture medium.

Colorectal cancer (CRC) is the world’s second leading cause of cancer-related mortality in men and women (Xi and Xu, 2021). Most CRC begin as benign adenomas that progress to aggressive carcinomas (Fumagalli et al., 2017). Adenomatous polyposis coli (APC) gene mutations in most CRC patients lead to the Wnt signaling pathway of tumorigenesis (Kleeman and Leedham, 2020). Most APC gene mutations occur in the β-catenin binding site, which results in β-catenin accumulation in the cytoplasm. Free β-catenin enters the nucleus and activates transcription factors, enabling the cells to acquire resistance to apoptotic stimuli (Kim et al., 2003; Jung and Park, 2020). CRC responds well to traditional chemotherapy, radiation therapy, and surgical resection treatments. However, previous treatments are inefficient for several reasons, including a lack of efficient molecular therapeutic targets or the existence of cancer stem cells (generate resistance to chemotherapeutic drugs/radiation) that metastasis or recurrence occurs (Sasaki and Clevers, 2018). Resistance to apoptosis is a hallmark of cancer and is associated with poor prognosis and tumor progression (De Toni et al., 2008). In preclinical models, resistance to apoptosis can be avoided by inducing apoptosis with death ligands from the TNF superfamily cytokines, such as TNF-α, FasL, and TRAIL (Van Geelen et al., 2004). Also, the heterogeneity of CRC tumors might explain differences in therapy sensitivity, resistance, and cancer growth patterns between individuals (Lannagan et al., 2021). Establishing advanced models that closely mirror the *in vivo* tumor structure and preserve the individual tumor features in a three-dimensional (3D) environment is required to develop effective preclinical strategies. Sato et al. developed an organoid culture method that allows for *ex vivo* long-term culture of stem cells or cancer cells in the 3D matrix (Sato et al., 2011). Tumor organoids cultured from fresh surgical resected tumors or needle biopsies are simplified versions of solid tumors as functionally versatile as cell lines, with 3D structure (Figure 1 A, B). Cancer cell lines and animal models like patient-derived tumor xenografts (PDTX) have traditionally been utilized in preclinical studies and have made significant progress in the development of cancer research (Kim et al., 2020). Due to the inefficiency of earlier cancer models in representing all aspects of primary tumors, many therapeutic agents used in these models are finally removed from clinical trials due to lack of effectiveness or unacceptable side effects (Xu et al., 2018; Veninga and Voest, 2021). Tumor organoids can be successful in establishing, cheaper to keep, and do not require animal models, which complies with ethical goals on animal welfare. This culture system also can be applied as a valuable platform in personalized medicine (Sasaki and Clevers, 2018).

Culture medium harvested from cultured cells, also known as a conditioned medium (CM), includes various small molecules, growth factors, and other metabolites secreted from cells. Various cell-derived components are present in CM for the appropriate culture time (Ueda et al., 2020). In this study, patient-derived colon cancer organoids were cultured and characterized. Then activated NK cells-conditioned medium (NK-CM) containing a complex of NK-released soluble apoptosis-inducing ligands and secreted factors consisting of IFN-γ and TNF-α that were considered for induction of apoptosis was investigated.

## Materials and Methods

### Tumor Specimen Collection

The Ethics Committee approved this study at the Tehran University of Medical Sciences (Code: IR. TUMS.VCR.REC.1398.881). Written consent was taken from the patients before their surgery. Resected tissue was taken from two colon cancer patients (75 years old female 48 years old male) performed at Sayyad-Shirazi University Hospital. The patients had not undergone any chemotherapy or radiation before surgery. The tumor tissue sample was transported in ice-cold DMEM/F12 complemented by penicillin-streptomycin, HEPES, and GlutaMAX.

### Primary Colon Tumor Cells and Crypt Isolation and Organoid Culture

Tumor tissue was processed under a biosafety hood within 1 hour of surgical resection. Non-epithelial tumor fragments, such as fat and muscle, should be removed from the culture (Driehuis et al., 2020). A scalpel was used to chop tumor tissue into smaller pieces of around 2–4 mm and then transferred into a 15 ml centrifuge tube. Tumor pieces were washed with ice-cold D-PBS (without Ca++ and Mg++) complemented by penicillin-streptomycin followed by at least 10 times gently pipetting up and down until supernatant was almost clear and then discarded. This process must be repeated several times. After washing, to isolate crypts and tumor cells, 10 ml enzyme-free gentle cell dissociation reagent (STEMCELL Technologies, Canada) was added and incubated on ice with medium speed (40 rpm) for 30 min. Then again, centrifuged at 5 min at 290 × g, 4 °C, and the supernatant was discarded. 5 ml ice-cold advanced DMEM/F12 supplemented with 1% bovine serum albumin (BSA) (all from Gibco) was added and vigorously pipetted to crypts, and tumor cells were removed from tumor pieces. Undigested tumor pieces were removed using a sterile 100 μm cell strainer, and crypts and tumor cells were obtained by centrifuging the cell suspension for 5 min at 290 × g, 4°C. The supernatant was carefully removed except for almost 100 µl. The resultant cell suspension in a ratio of 70 to 30 with 70% Matrigel [reduced growth factor, Phenol Red free (BD bioscience, United States)] is mixed, and 4,5 Matrigel domes (each dome 30 µl) were seeded on each well of 12 well culture plate. After complete polymerization of the Matrigel in a CO2 incubator (5% CO2, 37°C) for 10 min, 1 ml of basal culture medium (advanced DMEM/F12) with supplemented factors was added. Supplemented factors consist of 1% penicillin/streptomycin, 2 mM GlutaMAX, 10 mM HEPES (all from Gibco), 1.25 mM N-acetyl-cysteine (Sigma-Aldrich), 2 X B27 supplement (Gibco), 100 ng/ml human recombinant Noggin (Peprotech, United States), 50 ng/ml human recombinant epidermal growth factor (Invitrogen), 500 nM A83-01 (Tocris Bioscience, Bristol, United Kingdom), 10 nM gastrin (Sigma-Aldrich), and 10 μM SB202190 (Sigma-Aldrich). Fresh medium was added every 2 days. Wnt3A and the Wnt amplifier R-spondin1 are required supplements for producing organoids from normal epithelial cells (Sato et al., 2011). Wnt3A and R-spondin1 must be removed from the culture medium to selectively culture colon tumor organoids, avoiding normal colon organoid contamination. Tumor organoids were passaged every seven to 10 days. After carefully removing the culture medium without disturbing the Matrigel dome, 1 ml room temperature gentle cell dissociation reagent per well was added for organoid passage. There must be pipettes up and down several times after 1 minute to dissociate organoids. Dissociated organoids were removed from the culture plate and incubated with 2 ml gentle cell dissociation reagent for 10 min on a rocking platform with (40 rpm) speed at room temperature. After several pipettes up and down, the dissociated fragments are resuspended in fresh Matrigel and replated. The stem cells and transit-amplifying cells in organoid fragments will be formed mature organoid construction.

### Histopathology Assessment

#### Histochemistry

Organoids (seventh day of culture) were fixed in 10% neutral buffered formalin for 30 min at room temperature. Tumor tissue and organoids were dehydrated in a graded ethanol series followed by xylene for clearing and embedded in paraffin. Samples were cut into 4 µm thickness sections and stained with hematoxylin and eosin (H&E), alcian blue (AB), and periodic acid–Schiff (PAS) according to the standard protocol. Images were captured using an Olympus BX51 (Tokyo, Japan) microscope.

#### Immunohistochemistry

Paraffin-embedded tumor tissue and organoids were sectioned at a thickness of 4 µm. To deparaffinized and rehydrate the slides, sections were washed in xylenes and decreasing amounts of ethanol. These sections were heated for 10 min in 10-mM sodium citrate, 0.05% Tween 20, and pH 6.0 antigen-retrieval buffer. After cooled for 15 min, sections were incubated for 1 h in PBS with 0.5% Triton X-100 and 5% horse serum. The sections were incubated in blocking buffer with the associated primary antibody overnight at 4°C. Primary antibodies constitute the mouse anti-human Ki67 antibody, mouse anti-human intestinal alkaline phosphatase antibody, and anti-human CDX2 antibody (All from Invitrogen Corporation, CA, United States).

### *Ex Vivo* Expansion and Activation of Human NK Cell

NK cells were isolated using a human NK cell isolation kit (Miltenyi Biotech) and negative selection from buffy coats of healthy donors as directed by the manufacturer. Flow cytometry assessments of CD56^+^ and CD3^−^markers (FACSCalibur Becton Dickinson, United States) were used to characterize NK cells. MiltenyiBiotech’s NK Cell Activation/Expansion Kit was used to expand the NK cells. After the first 6 days, NK colonies were detected, and then every 3 days, a new RPMI 1640 medium and 10% FBS were given to the cells for 18 days, with IL-2 used for further activation (500 IU/ml) (Miltenyi Biotech).

### Flow Cytometry

NK cells were characterized using FITC anti-human CD3^−^markers and PE anti-human CD56^+^ (FACSCalibur Becton Dickinson). To further characterize IL-2-activated NK cells, CD69 and NKG2D were tested for cytotoxicity and activation. According to the manufacturer’s instructions, staining and flow cytometry were performed following the proliferation and activation processes. FlowJo 7 software was used to analyze the data (Tree Star, Inc.). Analyses were conducted in triplicate and quantified measures using GraphPad Prism 9.

### ELISA

Activated NK-CM was collected further IL-2-activation of NK cells, and a commercial ELISA kit (R&D Systems, Minneapolis, MN, United States) was used to assess the level of TNF-α and IFN-γ. All experiments were performed in triplicate. A wavelength of 450 nm was used to read the plate. The cytokine concentration (pg/ml) was determined using the standard curve. Fresh NK cells culture medium served as the control.

### Western Blotting

To investigate soluble apoptosis-inducing ligands in activated NK-CM, TRAIL, and FasL detected by western blotting. First, cell free-conditioned medium containing protease inhibitor (Thermo scientific) was loaded on 10% SDS-PAGE and subsequently transferred to a polyvinylidene difluoride (PVDF) membrane (Invitrogen, San Diego, CA). After 1 h of blocking with PBS containing 5% skim milk at room temperature, proteins were probed using the following antibodies: anti-TRAIL (1: 700, Abcam Cambridge United Kingdom) and anti-FasL (1: 300, Santa Cruz Biotechnology Inc.). Chemiluminescence detected proteins after incubation with HRP-labeled secondary antibodies (1:1,000 Santa Cruz Biotechnology Inc.). Analyses were conducted in triplicate and quantified measures using GraphPad Prism 9.

### Organoid Treatment With an Activated NK-CM

RPMI1640 medium supplemented with 10% FBS and IL-2 to investigate the NK cells culture medium effect in the organoid culture medium until 30% of the basal culture medium was replaced with NK cells culture medium. There was no morphologic change in size, shape, and structure of tumor organoids compared to the control group (100% DMEM/F12 as the basal culture medium). The IL-2-activated NK cells supernatant was collected and then centrifuged at 300 g for 10 min and filtered through a 0.22 µm filter to remove the cellular debris. The resultant conditioned medium was added to organoid culture as 30% of the basal culture medium.

### Annexin V-FITC Assay

The Annexin V-FITC kit (IQ Products, Groningen, Netherlands) was used to examine the induction of apoptosis in CRC organoids following 48 h of treatment with an activated NK-CM. Harvested organoids incubated with Annexin V-FITC before dissociation to single cells. Then organoids were dissociated using a gentle cell dissociation reagent into single cells at 37°C for 15 min. Cells were washed in calcium buffer, resuspended, and filtered through a 40 µm strainer. The analyses were carried out by a flow cytometer for apoptosis measurement (BD Accuri™ C6, BD Biosciences), and BD Accuri C6 software (BD Biosciences) was used for the data analysis. All treatments were carried out in duplicate.

### Statistical Analysis

Statistical analyses were performed using mean ± SEM with GraphPad Prism 9 software package (GraphPad Software, Inc., San Diego, United States). A *p*-Value of < 0.05 was considered statistically significant.

## Result

### Patient-Derived Colon Cancer Organoids Represent Patient Tumor Histological Characteristics

**FIGURE 2:**
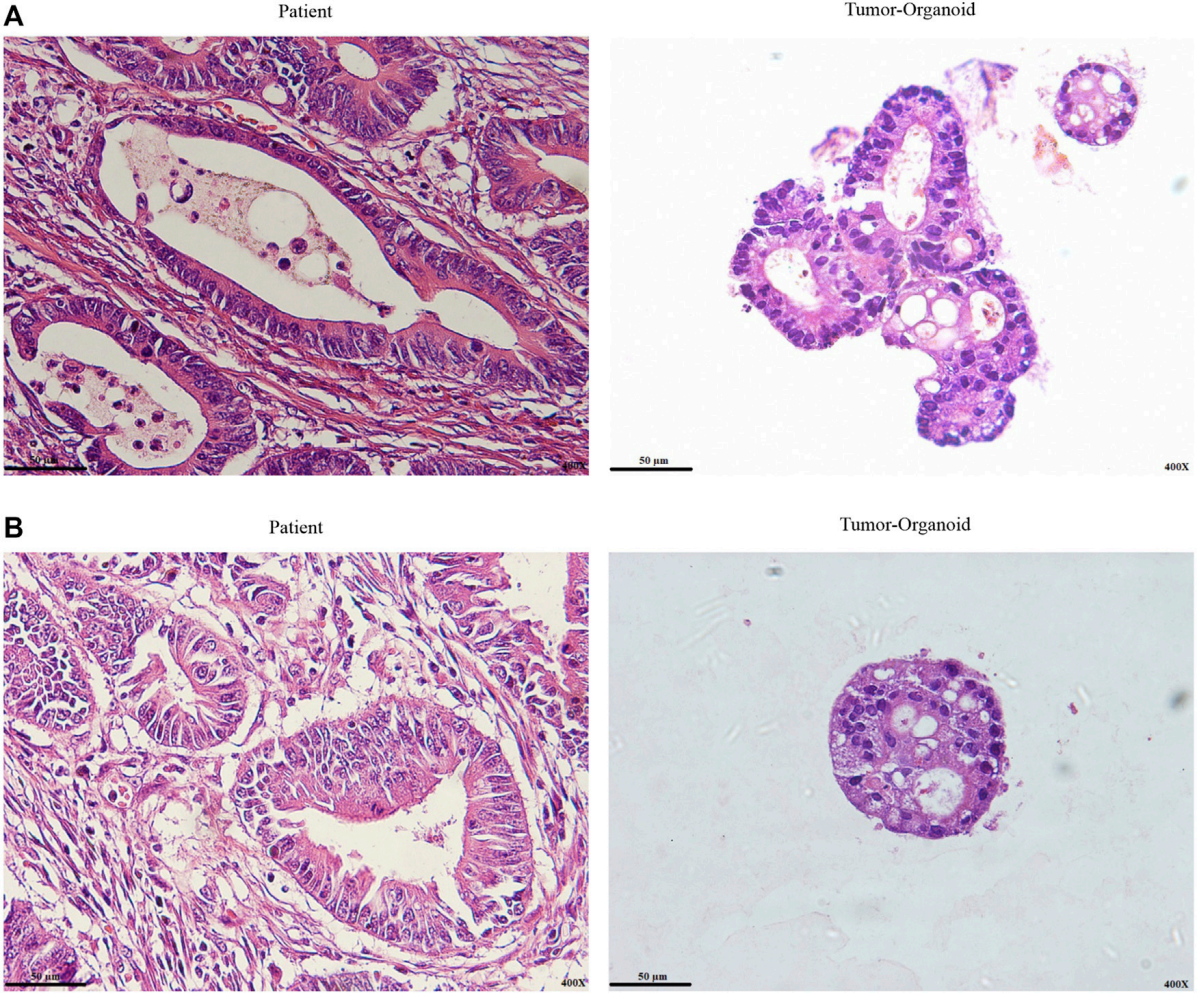
Representative H&E staining of colon cancer organoid and patient tumor. **(A)** Well-differentiated adenocarcinoma. **(B)** Moderately differentiated adenocarcinoma. Histological differentiation pattern analysis indicated tumor organoids retain patient tumor histopathological characteristics. In both paraffin-embedded sections from the original tumor and cultured organoids from the same patient, well-differentiated and moderately differentiated adenocarcinoma were seen. Scale bar: 50 μm, ×400 magnification.

Most colon cancer organoids grew without Wnt signaling ligands (Sato et al., 2011; Fujii et al., 2016). In the present study, we cultured patient-derived colon cancer organoids that are independent of Wnt amplifier factors. The pathologist’s expert performed a histological examination and diagnostic evaluation on the original tumor sample and tumor organoids on paraffin-embedded sections to determine whether the histological features of the source tumors were retained in the colon cancer organoids. We observed well-differentiated and moderately differentiated adenocarcinoma in one case in patient tumor sections. As expected, tumor organoids derived from the same patient represent both differentiation patterns (Figures 2A,B).

### The Status of Colon Cancer Organoids Differentiation

**FIGURE 3:**
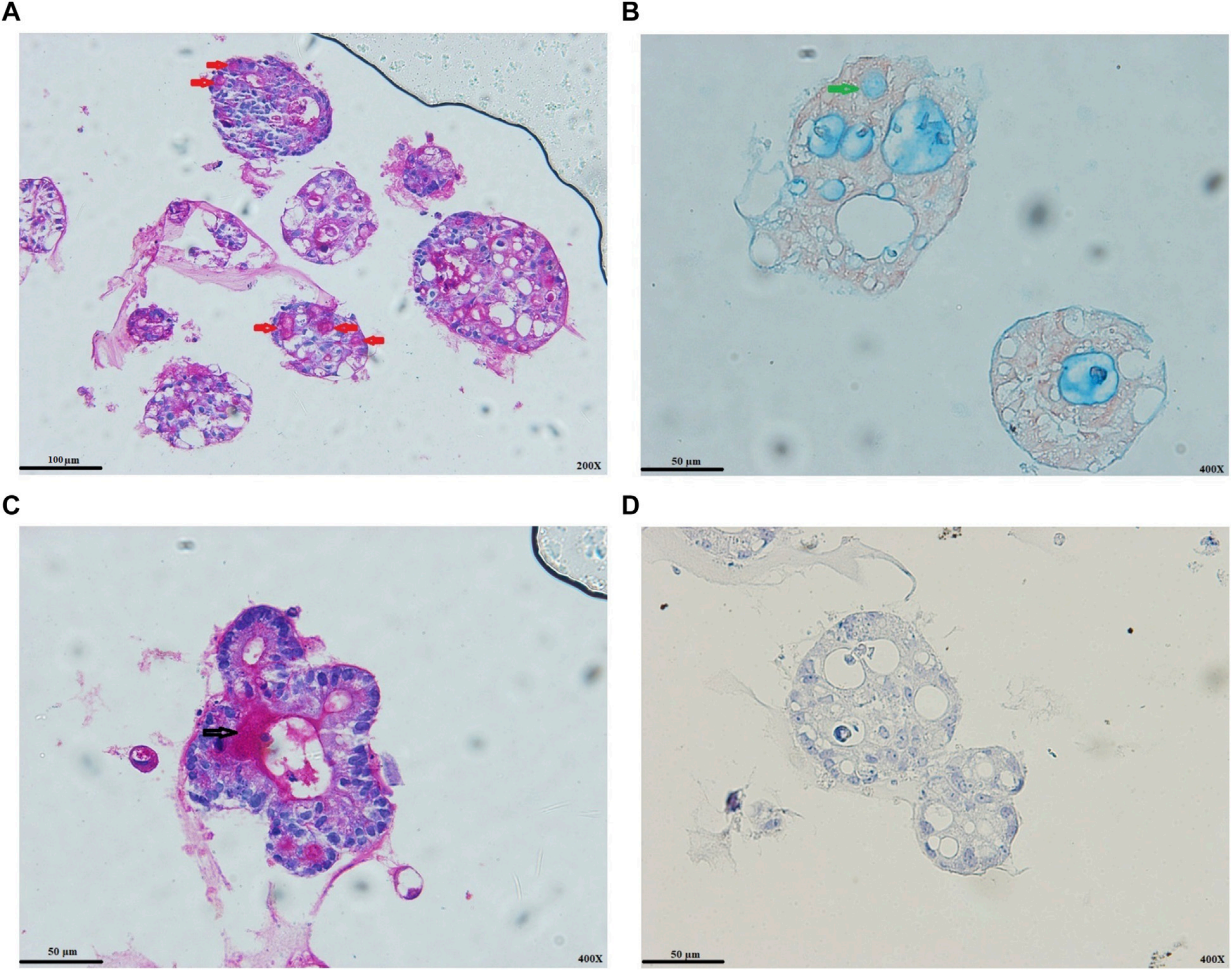
Colon cancer organoids differentiation status. **(A)** PAS-positive goblet cells (red arrows). **(B)** AB-positive goblet cell (green arrows). **(C)** Secretion process of goblet cell mucus into the lumens of organoids (black arrow). **(D)** IHC staining for alkaline phosphatase. As expected, Wnt activity strongly inhibits enterocyte differentiation. Scale bar: 50 μm and 100 μm, ×400 and ×200 magnification.

Goblet cells are distributed from the proximal small intestine with increasing proportion to the distal colon (Múnera et al., 2017). PAS staining represented the goblet cells’ differentiation status at the protein level. Mucins in tumor organoids’ lumens and goblet cells reacted strongly with the PAS (Figure 3A), validated by AB staining (Figure 3B). We also observed the secretion process of goblet cell mucus into the lumens of the organoids (Figure 3C). In most colon cancer, Wnt signaling activity has a strong effect of inhibition on enterocyte differentiation (Sato et al., 2011). As expected, despite observing AB/PAS-positive goblet cells, we could not observe the alkaline phosphatase staining for colon cancer organoids (Figure 3D).

### Colon Cancer Organoids Preserve the Histopathological Characteristics of the Patient Tumor

**FIGURE 4:**
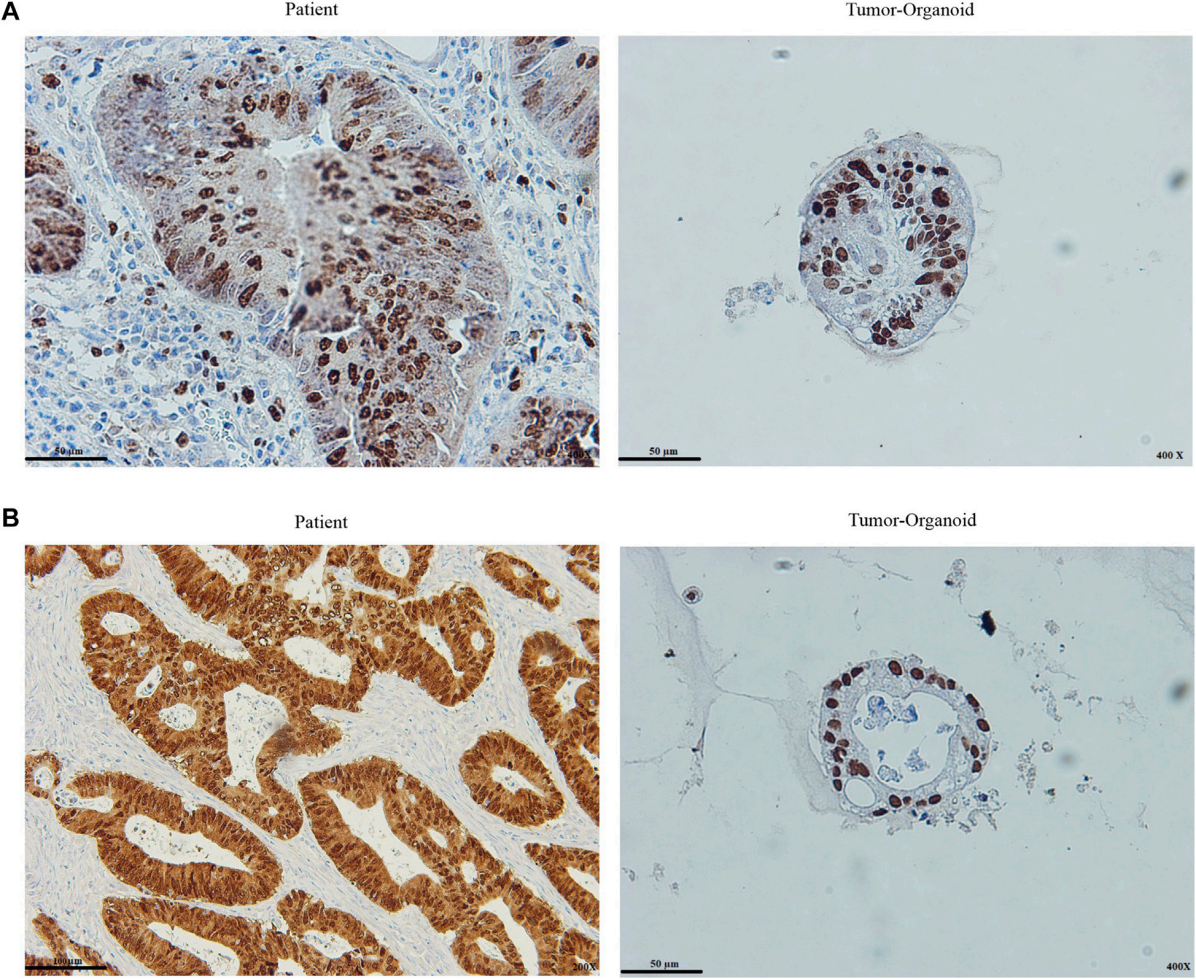
Histopathological assessment of patient tumor and tumor organoid **(A)** Ki67 staining of the colon cancer organoids and original tumor **(B)** CDX2 staining of the colon cancer organoids and original tumor. Both stain intensity and presence quantified ki67 and CDX2. Intensity defines staining strength, and presence is expressed as the percentage of cells with staining. The staining intensity is evaluated within the nuclear fraction. Scale bar: 50 μm and 100 μm, ×200 and ×400 magnification.

Caudal type homeobox 2 (CDX2) is a major regulator of intestinal formation and differentiation, and also its expression is peculiar to the intestinal epithelium (Salari et al., 2012). Colon tumors that lack CDX2 expression are more prone to have aggressive characteristics such as advanced stage, poor differentiation, vascularization, BRAF gene mutation, and the CpG island methylator profile (Dawson et al., 2014). Cell proliferation is associated with Ki67 expression. Ki67 levels were substantially related to lymph node metastases and a poor prognosis for CRC (Li et al., 2016). The presence and intensity of stain expression of Ki67 and CDX2 markers were graded in this investigation. The tumor organoid and the patient tumor were significantly positive for the Ki67 and CDX2 markers. As previously described (Ganesh et al., 2019), quantified presence is taken into account: percentage of cells stained: 1 = 30%, 2 = 30–60%, 3 = > 60%, and intensity: staining strength: 1 = mild, 2 = moderate, 3 = high. In comparison to tumor tissue, tumor organoids preserved similar levels of intensity and presence of the markers, indicating the origins of the tumors (Figures 4A,B).

### Immunophenotyping of NK Cells

**FIGURE 5:**
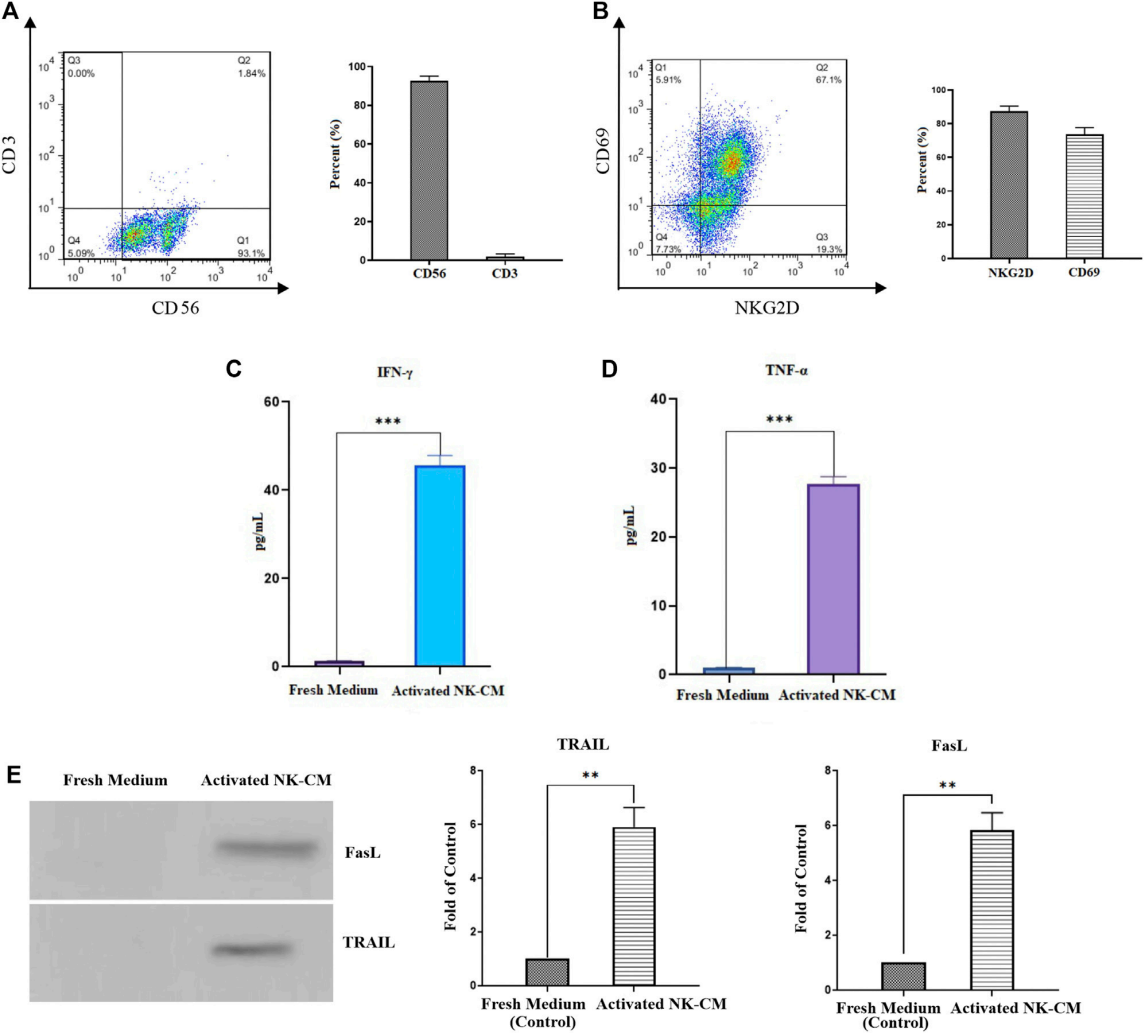
Characterization and activation status of NK cells. **(A)** The population of CD56 + CD3-NK cells after MACS isolation. **(B)** The activation status of IL-2-activated NK cells by NKG2D and CD69 markers. **(C)** The IFN-γ concentration of IL-2-activated NK cells conditioned medium (NK-CM). **(D)** The TNF-α concentration of IL-2-activated NK-CM. The immunostimulatory cytokines level of IL-2-activated NK-CM was significantly increased than the control group (fresh NK cells culture medium). **(E)** Soluble FasL and TRAIL are present in activated NK-CM determined by western blotting. Data presented as means ± standard error (M± SE). Statistical significance was determined using an unpaired *t*-test with *p < 0.05. ns: non-significant.

Isolated and expanded NK cells were identified by the presence of CD56 and the lack of the CD3 marker. According to flow cytometric analysis, the purity of NK cells was 93.1%, which comprised CD3^−^and CD56 ^+^ cells (Figure 5A). NK cells freshly isolated were cultured and expanded for 18 days in the presence of IL-2. Immunophenotyping of NKG2D and CD69 further investigated the activation and cytotoxicity capabilities. More than 86% of IL-2-stimulated NK cells were positive for NKG2D and 73% for CD69 (Figure 5B).

### Activated NK-CM Contains Cytokines and Soluble Death Ligands

The antitumor effector status of NK cells is assessed by measurement of IFN-γ and TNF-α cytokines secretion (Jiang et al., 2014). IFN-γ has been shown to increase the production of death ligands on immune cells, with TRAIL expression in NK cells being particularly affected (Smyth et al., 2001). According to the ELISA results, the levels of IFN-γ and TNF-α in the activated NK-CM were significantly higher than in the fresh CM. (Figures 5C,D). Western blot assessments showed that the soluble variant of FasL and TRAIL is detectable and present in activated NK cells-CM. (Figure 5E).

### Activated NK-CM Has No Significant Effect on Apoptosis Induction

**FIGURE 6:**
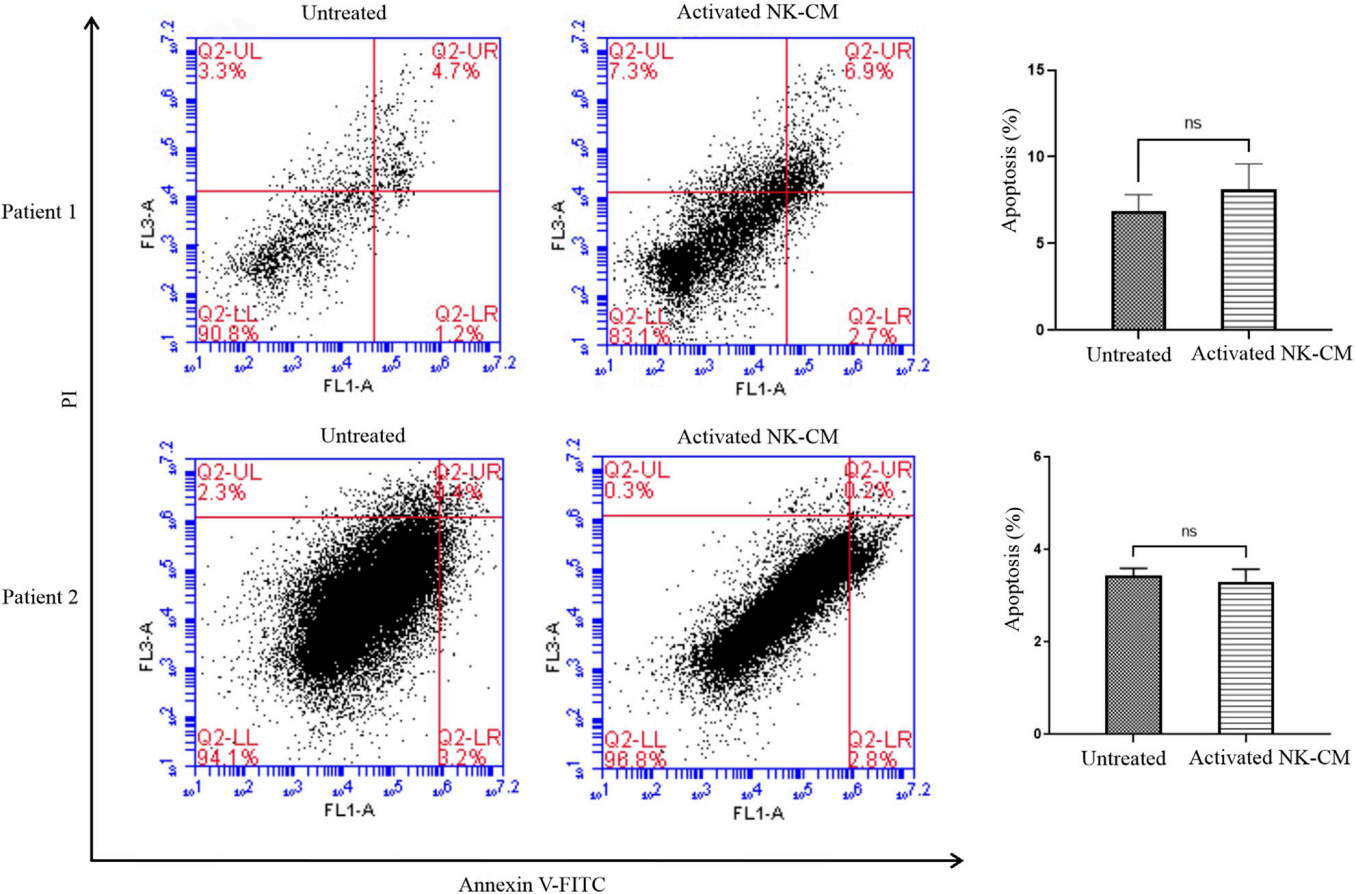
Annexin V-FITC/propidium iodide (PI) double staining assay. Colon tumor organoids were harvested after 48 h treatment with IL-2-activated NK-CM, then were incubated with Annexin V FITC for 20 min and dissociated. PI was added just before the flow cytometry analysis. The percentage of Annexin V-FITC-positive/PI-negative and Annexin V-FITC-positive/PI-positive cells are also shown. Data presented as means ± standard error (M± SE). Statistical significance was determined using an unpaired *t*-test with *p < 0.05.

Flow cytometry analysis was performed to analyze the percentage of apoptotic and non-apoptotic cells staining tumor organoids with Annexin V-FITC/PI. There was no significant increase in the early apoptotic rate (Annexin V-FITC-positive/PI-negative) and late-stage apoptotic/necrotic cells (Annexin V-FITC-positive/PI-positive). (Figure 6).

## Discussion

In CRC, an imbalance in both cell death and cell renewal with enhanced proliferation is a known mechanism for the development of tumorigenesis. It has been shown apoptosis resistance appears when the colonocyte develops from normal epithelium to carcinoma (Huerta et al., 2006). Conventional cancer models cannot accurately describe the underlying pathways important to immune cell-based therapies for solid tumors. Patient-derived colon cancer organoids are a reliable model for studying cancer that can replace and reduce animal use in preclinical research. Patient-tumor modeling might be hampered by intratumoral heterogeneity because the tumor sample is a minuscule portion of a much larger tumor (Short et al., 2017). We established tumor organoids with varied differentiation patterns by sampling different parts of the patient’s tumor. Also, CDX2 and Ki67 expressions were compared between tumor organoids and patient tumor sections. The results show a high proliferation status with more than 60% expression level of Ki67 was retained in patient-derived colon cancer organoids. CDX2, a CRC-specific nuclear marker in almost 100% of colon tumor organoids, was expressed. These data suggest that, in contrast to 2D cell culture, organoids effectively reflect the distinct aspects of the individual tumor and retain the tumor’s 3D structure. Moreover, they are appropriate preclinical models based on histological analyses for personalized settings.

NK cells express both soluble and membrane-bound FasL and TRAIL (Diaz Arguello and Haisma, 2021). We showed soluble FasL and TRAIL are present in IL-2 activated NK-CM. Also, it has been verified that activated NK cells secret immunostimulatory cytokines, including TNF-α and IFN-γ, that are needed for apoptosis induction and sensitizing DRs of cancer cells, respectively. So, the question in our investigation is whether an activated NK-CM containing IFN-γ and soluble apoptosis-inducing ligands as protein can induce apoptosis of colon cancer organoids or not a therapeutic supplement.

In this work, we treated patient-derived colon cancer organoids with IL-2 activated NK-CM (30% final concentration) to explore apoptosis induction. Flow cytometry analysis has shown no significant change in apoptosis induction. These findings indicate that soluble apoptosis-inducing ligands along with IFN-γ are unlikely to lead to apoptosis. Another investigation has proven soluble counterparts of FasL and TRAIL. However, they bind to their cognate receptors but should not or minimally trigger these receptors’ apoptosis signal transmission (Berg et al., 2007). Several factors involved in TRAIL signaling make cancer cells resistant to inducing apoptosis by TRAIL. It has been shown soluble TRAIL can bind to decoy receptors (DcR1, DcR2, and OPG) that do not activate apoptosis (O'Leary et al., 2016). In a study conducted by Setroikromo et al., using a soluble variant of TRAIL on CRC cells, it was shown that DR5 was expressed on extracellular vesicles (EVs) secreted by CRC cells. The DR5-coated EVs act as a decoy receptor by competing with target cell DR5 to bind TRAIL. It reduces effective TRAIL binding and, as a result, resistance of CRC cells to TRAIL-related apoptosis induction (Setroikromo et al., 2020). Pavet et al., in 2010, reported that DR5-selective TRAIL-mimetic peptides (M1d) have vigorous tumoricidal activity against xenografted colon cancer cells (Pavet et al., 2010). A recent study demonstrated that DR5 activation independently of DR4 and DcR2 can promote TRAIL-induced pro-apoptotic and pro-survival signals (Shlyakhtina et al., 2017). Another study demonstrated recombinant soluble TRAIL has a proliferative impact on neuroblastoma cells. This effect contrasts with the well-demonstrated involvement of membrane-bound TRAIL of NK cells in cancer cell apoptosis (Sheard et al., 2013).

It has been shown in contrast to transmembrane FasL that it powerfully stimulates Fas, but its soluble form, while capable of receptor binding, does not activate Fas but acts as a competitive inhibitor of transmembrane FasL (Bremer et al., 2009). It has also been reported Fas-mediated apoptosis is not effective in most colon cancer cell lines, suggesting that the Fas-mediated apoptosis signaling pathway is dysfunctional. In addition, CRC cells can “counterattack and kill immune cells” by presenting FasL to lymphocytes with functional Fas and activation pathways (Kim et al., 2003). Many other strategies for disruption of Fas-mediated apoptosis have been discovered in tumors. It has been proposed that p53 mutations, downregulation of Bak, Bax, and overexpression of Bcl-2 are possible candidates for colon cancer cell resistance to Fas-mediated apoptosis (O'Connell et al., 2000).

In conclusion, the findings indicate that activated NK cells release the soluble form of apoptosis-inducing ligands in the culture medium in addition to IFN-γ secretion. Also, recruiting activated NK-CM against patient-derived colon cancer organoids does not affect the induction of apoptosis. However, our study confirms that the organoid culture system supports translational cancer research as an *ex vivo* test platform.

## Data Availability

The raw data supporting the conclusion of this article will be made available by the authors, without undue reservation.
